# 

**Published:** 1842-12-17

**Authors:** 


Published EVERY SATURDAY by J. C. TURNPENNY, N. E. corner of Spruce and Tenth streets.

*Price of Subscription*—Three Dollars a year, or two copies for Five Dollars to the same post office, always in advance. Subscriptions must commence with the beginning of the annual volume. Letters to be addressed, post free, to the Editors of the Medical Examiner.

*Rates of Advertising in the Medical Examiner*—For one insertion of a square of twelve lines, one dollar, and for each additional insertion, half a dollar.

